# Cross-Lagged Panel Networks of Distinct Complex Post-Traumatic Stress Disorder Symptom Trajectories Among Young Adults With Adverse Childhood Experiences

**DOI:** 10.1155/da/8823021

**Published:** 2025-09-16

**Authors:** Aiyi Liu, Liying Zhang, Mingxiao Liu, Wang Ziwei, Xinchun Wu

**Affiliations:** ^1^State Key Laboratory of Cognitive Science and Mental Health, Institute of Psychology, Chinese Academy of Sciences, Beijing, China; ^2^Department of Psychology, University of Chinese Academy of Sciences, Beijing, China; ^3^Beijing Key Laboratory of Applied Experimental Psychology, National Demonstration Center for Experimental Psychology Education (Beijing Normal University), Faculty of Psychology, Beijing Normal University, Beijing, China

**Keywords:** ACEs, CPTSD symptoms, cross-lagged panel network, trajectories, young adults

## Abstract

**Background and Objectives:** Young adults with a history of adverse childhood experiences (ACEs) may exhibit varying trajectories of complex post-traumatic stress disorder (CPTSD) symptoms over time. Unraveling the patterns of interactions between CPTSD symptoms across distinct trajectories is crucial. This study aimed to investigate the longitudinal relationships, changes, and central symptoms in CPTSD networks over time across distinct CPTSD trajectory groups.

**Methods:** This longitudinal study followed 1277 university students (47.5% male) who reported ACEs from China through three waves of surveys. ACEs were assessed at baseline, while symptoms of CPTSD were measured at all three time points. Growth mixture modeling (GMM) was used to identify CPTSD symptom trajectories, and cross-lagged panel network (CLPN) analysis estimated the longitudinal relationships among CPTSD symptoms within these trajectories.

**Results:** Two distinct and consistent CPTSD symptom trajectories were identified: a high-risk group and a resistance group. In the high-risk group, “disturbed relationships” (DRs) and “negative self-concept” (NSC) emerged as the strongest predictors of other symptoms at various time points. In the resilient group, the predictive influence of DR and NSC on other symptoms was attenuated. Instead, “affective dysregulation” (AD) emerged as the central symptom, demonstrating the strong predictive associations with other symptom domains.

**Conclusions:** These findings reveal directional relationships among symptoms in young adults. Symptoms related to disturbances in self-organization (DSO), identified through centrality indices, are key drivers of symptom network development in different CPTSD trajectories. Targeting these symptoms in interventions for young adults with ACEs may help prevent or reduce CPTSD progression.

## 1. Introduction

Globally, about 10%–30% of children and adolescents experience adverse childhood experiences (ACEs; [1]), which include physical, emotional, and sexual abuse, neglect, and family disruptions before the age of 18, such as witnessing domestic violence, substance abuse, mental illness, or criminal behavior [2]. ACEs are linked to mental health challenges [3] and can increase the risk of adult mental illness, depending on the type and severity of the trauma [4, 5]. Emerging adulthood, which includes the typical college years, is a critical developmental stage involving identity formation and major life transitions [6]. For individuals with a history of ACEs, this period may reactivate unresolved trauma and increase vulnerability to psychological difficulties. College students, in particular, often face academic, social, and environmental stressors, further elevating this risk [7]. Recent studies have also highlighted a strong link between ACEs and mental health problems in this population [8, 9].

The eleventh version of the International Classification of Diseases (ICD-11, [10]) classifies complex post-traumatic stress disorder (CPTSD) under the category of stress-associated disorders. CPTSD consists of three PTSD clusters: (1) re-experiencing (RE) the trauma in the here and now, (2) avoidance (AV) of traumatic reminders, and (3) a persistent sense of current threat (TH) that is manifested by exaggerated startle response and hypervigilance, as well as three additional clusters that reflect “disturbances in self-organization” (DSO): (1) affective dysregulation (AD), (2) negative self-concept (NSC), and (3) disturbances in relationships. CPTSD often arises from prolonged, repetitive, or multiple forms of trauma exposure, especially interpersonal traumas [5]. The trauma leading to CPTSD under the ICD-11 framework can be triggered by a traumatic event or series of traumatic events [11]. Kira [12] categorizes trauma into three types: Type I trauma involves a single, isolated event, such as a car accident. Type II traumas are a series of related events that occurred over a period and then ceased, such as ACEs in adulthood. Type III traumas (continuous traumatic stress) involve ongoing exposure, such as current abuse, neglect, or domestic violence. Individuals exposed to Types II and III traumas often cannot escape during the events, and repeated, interpersonal traumas are recognized as particularly potent contributors to CPTSD [11, 13–15]. While CPTSD may result from various traumatic experiences, childhood maltreatment is uniquely impactful due to its early disruption of emotional, cognitive, and relational development [16, 17]. Empirical studies have demonstrated that CPTSD is associated with greater functional impairment, higher comorbidity with mood and anxiety disorders, and poorer treatment response compared to PTSD [18]. In addition, CPTSD is associated with more severe trauma-related symptoms and adverse outcomes, including addictive behaviors [19], psychotic-like experiences such as auditory hallucinations, paranoid ideation, and passivity phenomena [20], and suicidal behaviors [21]. Therefore, focusing on individuals with ACEs offers unique theoretical and clinical insights into the development and expression of CPTSD.

From a developmental psychopathology perspective [22], individuals exposed to similar early adversities may follow divergent psychological trajectories. These differences are shaped by variations in biological, psychological, and social-contextual systems that influence adaptation across development. As individuals grow, the presence of protective factors and the accumulation of risk factors can direct their developmental pathways toward either resilience or maladaptation. Accordingly, individuals with a history of ACE may exhibit heterogeneous trajectories of CPTSD symptoms over time [23]. Recent longitudinal studies have examined the predictors, mechanisms, and structural stability of CPTSD symptomatology [24–26]. However, these studies have primarily adopted a variable-centered approach.

A developmental theory of post-traumatic psychological responses emphasizes that, in the context of childhood adversity, psychological symptoms may exhibit a certain degree of continuity over time. However, the expression of this continuity is often heterogeneous [27, 28]. Person-centered trajectory modeling approaches that account for individual differences in estimating symptom patterns can provide a more precise understanding of the developmental course of trauma-related psychopathology following childhood adversity [29]. Currently, only one longitudinal study with a 6-month interval has explored the trajectories of CPTSD in a sample of 294 college students who experienced ACEs. The results identified three subgroups with different levels of symptoms: the low-symptom group, the moderate-symptom group, and the high-risk group [30]. In addition, several long-term follow-up studies on PTSD have provided valuable insights into symptom trajectories. According to the developmental model of continuity and change in PTSD symptoms, PTSD does not represent a fixed or uniform response to trauma. Rather, individuals may exhibit diverse developmental trajectories, including initial adaptation, delayed onset, symptom exacerbation, and spontaneous recovery. These variations are shaped by individual differences in trauma exposure, psychological vulnerability, and resilience mechanisms [27]. For example, a longitudinal study of Chinese adolescents exposed to the Wenchuan earthquake identified five distinct patterns of psychological response: resistance, recovery, relapsing/remitting, delayed dysfunction, and chronic dysfunction [31]. Similarly, a 7-year investigation involving 55,632 Japanese disaster responders revealed five PTSD symptom trajectories: resilient, recovery, incomplete recovery, late-onset, and chronic severe [32].

In summary, previous research has emphasized the importance and necessity of investigating CPTSD symptom trajectories. Such an approach is particularly valuable for identifying high-risk subgroups. However, to further improve the precision of interventions, a deeper understanding of the specific symptom structures within these at-risk groups, as well as the relationships among these symptoms, is crucial. This approach has further strengthened our understanding of the dynamic interactions between symptoms over time, highlighting how certain symptoms may serve as central drivers within the symptom network [33–35]. Network analysis is a widely used method in psychopathology, uncovering direct interactions among symptoms of psychiatric disorders and visualizing them as network graphs [36–38]. These network graphs consist of two main components: nodes, which represent individual symptoms and are illustrated as circles, and edges, shown as lines connecting nodes to indicate the relationships between symptoms [37]. This approach conceptualizes psychiatric disorders as intricate networks of interdependent symptoms that reinforce one another [36]. Notably, network analysis can identify the most influential symptoms within a network, offering valuable insights into their dynamic interactions [39, 40]. Several studies have employed network analysis to explore various aspects of CPTSD, such as its structural validity [41, 42], symptom interconnections, central symptoms across different trauma contexts [43], and its relationship with other psychiatric disorders and comorbid symptoms [44–46]. These investigations have provided valuable insights into the evolution of CPTSD symptoms in individuals exposed to ACEs. However, previous studies in this area have predominantly relied on cross-sectional network analyses, which are limited in their ability to capture the temporal dynamics and directionality of symptom interactions. Although Bayesian network analysis offers a promising approach by inferring probabilistic dependencies and potential causal directions from cross-sectional data [47], it still cannot fully address the limitations inherent to nonlongitudinal designs, particularly when it comes to establishing temporal precedence and within-person symptom fluctuations. Therefore, longitudinal approaches remain essential for accurately modeling the dynamic interplay of symptoms over time.

Cross-lagged panel network (CLPN) analysis offers a powerful and innovative method for modeling the dynamic temporal relationships among psychological symptoms [40]. In contrast to traditional longitudinal models, CLPN simultaneously captures autoregressive and cross-lagged associations among multiple variables, enabling researchers to investigate how specific symptoms at one time point predict changes in other symptoms at subsequent time points [48]. This approach is particularly valuable in the study of complex disorders such as CPTSD, where symptom domains often interact in self-reinforcing patterns over time. A unique feature of CLPN is its ability to estimate out-expected influence (out-EI) and in-expected influence (in-EI) for each symptom node. Out-EI reflects the degree to which a symptom predicts changes in other symptoms over time, thereby indicating its potential role in driving the network. In contrast, in-EI reflects the extent to which a symptom is influenced by others, signaling its sensitivity to changes within the system. These metrics help identify core symptoms that may serve as promising targets for intervention or theoretical modeling [49]. For example, a recent CLPN study on CPTSD symptoms found that NSC exhibited the highest out-EI, suggesting a potential causal role in sustaining symptom networks. In comparison, AV symptoms showed the highest in-EI, indicating greater vulnerability to influence from other symptoms [50]. Overall, CLPN provides both methodological and practical advantages for capturing the temporal dynamics of CPTSD and informing early intervention strategies.

The first objective of this study is to investigate the trajectories of CPTSD symptoms in young adults with a history of ACEs, aiming to identify high-risk subgroups. The second objective is to conduct an exploratory analysis of networks across different CPTSD trajectories. By calculating symptom centrality indices, we aim to identify central symptoms that are more likely to activate and predict other symptoms, thereby providing insights for targeted interventions to address CPTSD symptoms in young adults with a history of ACEs.

## 2. Methods

### 2.1. Participant and Procedure

Data were collected at the beginning of each academic year from universities in the Hubei, Guizhou, and Guangdong provinces of China. During a mental health education course, students completed an online questionnaire via the “Wenjuanxing” platform within a standardized time frame during class. There were no missing values since incomplete online questionnaires could not be submitted. Participation was entirely voluntary, and respondents were free to decline or withdraw from the survey at any time without any negative consequences. The study was conducted by three Ph.D. students trained in clinical and counseling psychology, with school psychological teachers available for follow-up counseling if requested. It received approval from the research ethics committee of the first author's institution (Approval Number: 202003190026).

This study was part of a longitudinal research project entitled *“*The Effects of Childhood Interpersonal Trauma on Post-Traumatic Psychological Adjustment Among College Students,” aimed at investigating the long-term psychological effects of childhood interpersonal trauma. Data were collected at three time points: September 2021 (T1), September 2022 (T2), and September 2023 (T3), each aligned with the beginning of the academic year to ensure consistency and minimize disruption from exams or breaks. Of the 4335 college students with reported ACEs who participated in T1, 2306 were retained at T2, and 1277 completed all three waves. To maintain analytic consistency, only those with complete data across all waves were included in the final sample. The final analytic sample consisted of 1277 students, with a mean age of 20.34 years (SD = 2.80). Among them, 607 (47.5%) identified as male and 670 (52.5%) as female. Descriptive statistics on the prevalence of ACEs and negative life experiences are presented in Table 1.

### 2.2. Measures

#### 2.2.1. ACEs

This study used the ACEs questionnaire to identify family adversity [2, 51]. The questionnaire comprised 29 items designed to evaluate different forms of abuse (physical, sexual, and emotional), neglect (physical and emotional), and household dysfunction (parents' divorce or separation, household violence, substance abuse by family members, mental illness, or incarceration). Participants rated ACE items to indicate the extent to which each item applied to them. In this study, all participants completed the ACEs inventory at the baseline (T1) assessment.

#### 2.2.2. Lifetime Traumatic Exposure

Lifetime trauma exposure was measured using a modified version of the Chinese life events checklist for DSM-5 (LEC-5; [52, 53]), which assesses exposure to a range of potentially traumatic events. The standard LEC-5 consists of 17 items covering a broad range of trauma types. Participants were asked to indicate their exposure to each event using multiple response options: “happened to me,” “witnessed it,” “learned about it,” “part of my job,” “not sure,” and “does not apply.” A traumatic event was considered endorsed only if the participant selected “happened to me” or “witnessed it.” To reduce participant burden and minimize conceptual overlap with the ACEs questionnaire, several redundant items were removed. The final version included 12 items and was used for all analyses. The validity of this version of the questionnaire has been established in Chinese college student populations [54]. In this study, all participants completed the LEC-5 at the baseline (T1) assessment.

#### 2.2.3. International Trauma Questionnaire (ITQ)

The ITQ [55] was developed to assess the diagnostic criteria for CPTSD as defined by the ICD-11. The ITQ includes six PTSD items and six DSO items. The PTSD symptom clusters of RE, AV, and sense of TH are measured using two items each. Three items measure functional impairment associated with these symptoms. The DSO symptom clusters of AD, NSC, and disturbances in relationships are measured by two items each. Each one of the conditions is also assessed by three items addressing functional impairment. The ITQ has been thoroughly validated among various trauma-exposed populations in China [16, 56]. In this study, the ITQ demonstrated strong internal consistency, with Cronbach's *α* coefficients for PTSD symptoms at T1, T2, and T3 reported as 0.94, 0.93, and 0.93, respectively.

### 2.3. Statistical Analyses

#### 2.3.1. Trajectories of CPTSD Symptoms

We employed growth mixture modeling (GMM) to examine the longitudinal trajectories of CPTSD symptoms. GMM allows for the identification of distinct classes with varying patterns of change over time based on participants' temporal variations [57]. This method categorizes participants into homogeneous subgroups within each class, while allowing for heterogeneous differences between classes. GMM requires prespecification of the number of classes. In this study, we incrementally increased the number of classes, adding one class at a time, and compared model fit indices across models to identify the best-fitting model. For each classification, the estimation was repeated using 200 random initial values to avoid spurious convergence to a local maximum. In accordance with Wang and Wang's [58] guidelines, the model fit of the GMM included the Akaike Information Criterion (AIC), the sample size-adjusted Bayesian Information Criterion (aBIC), entropy, posterior probabilities for each class, the Lo–Mendell–Rubin adjusted likelihood ratio test (LMR–LRT), and the bootstrapped likelihood ratio test (BLRT). Lower values for AIC and aBIC indicate an improved model fit, while higher entropy reflects more precise class classification. Furthermore, subgroups comprising less than 5% of the sample were considered potentially redundant [59]. Significant results for the LMR–LRT and BLRT in a K-class model indicate that the K-class model offers a superior fit compared to the K-1 class model.

#### 2.3.2. CLPN

The construct CLPNs were calculated using the R package glmnet [40, 60]. We applied the least absolute shrinkage and selection operator (LASSO) with 10-fold cross-validation to refine parameter selection, minimizing minor regression coefficients to zero [61]. In these CLPN graphs, nodes represent symptoms, line thickness indicates the strength of associations, and arrows show the direction and magnitude of cross-lagged effects.

The centrality indices for in-EI and out-EI were computed using the qgraph package in R [61]. The in-EI represents the sum of all incoming edge values linked to a specific symptom, indicating the extent to which baseline nodes account for the variance in the symptom observed at follow-up. Conversely, out-EI is derived by summing the values of outgoing edges from a given symptom, thereby reflecting the influence that the baseline symptom exerts on other follow-up nodes [40, 48]. Compared to strength centrality, which aggregates absolute edge weights regardless of direction, expected influence retains the sign of the associations (positive or negative). This distinction is particularly important in psychopathology networks, where both inhibitory and facilitative symptom pathways may exist. Therefore, expected influence provides a more nuanced and clinically meaningful index of symptom centrality [62].

The reliability and stability of the CLPNs were evaluated using multiple procedures provided by the R package *bootnet* [63]. To begin, we assessed the accuracy of the edge weights by constructing 95% confidence intervals (CIs) for each edge, utilizing nonparametric bootstrapping with 1000 iterations [48, 63]. Following this, we used case-drop bootstrapping to calculate correlation stability (CS) coefficients, which allowed us to evaluate the consistency of the centrality indices' rank order. The CS coefficient ranges from 0 to 1, with values exceeding 0.25 indicating an acceptable level of stability [48, 63]. Lastly, we examined the statistical significance of differences in edge weights to ensure they were meaningfully distinct.

## 3. Results

### 3.1. Descriptive Statistics and Correlations

Table 1 presents the prevalence of ACEs and negative life events among college students with a history of ACEs. The most frequently reported ACEs were mother treated violently, parental divorce/separation, and physical neglect. Among negative life events, the most prevalent were any other stressful event or experience, natural disaster, and physical assault.  Table 2 summarizes the descriptive statistics and bivariate correlations among the main study variables. Gender was significantly correlated with CPTSD symptoms at T3, and age was negatively associated with CPTSD symptoms at both T1 and T2. Negative life events were positively correlated with CPTSD symptoms at T1 and T2. Furthermore, CPTSD symptoms across the three time points were significantly correlated, indicating temporal stability of symptom severity. Descriptive statistics for individual CPTSD symptoms at each time point, stratified by group, are presented in Table S1.

### 3.2. Trajectories of CPTSD Symptoms

Table 3 presents the fit indices for the candidate 1-class to 6-class GMMs examining CPTSD symptom trajectories. Overall, both AIC and aBIC values decreased with the addition of more classes, suggesting improved model fit. Notably, the 2-class solution demonstrated a substantial reduction in AIC (9941.548) and aBIC (9963.281) compared to the 1-class model, indicating a significantly better fit. Although models with more than two classes exhibited continued declines in AIC and aBIC, the magnitude of improvement diminished progressively, and concerns regarding interpretability emerged. Entropy values increased across models, peaking at 0.960 in the 6-class solution. However, the 6-class model failed to yield a viable smallest class (0%), indicating overfitting and reduced classification utility. Similarly, the 5-class and 3-class solutions included classes with proportions below the commonly accepted threshold of 5% for reliable interpretation [59]. In contrast, the 2-class model yielded high classification accuracy (entropy = 0.897), with the smallest class comprising 22.63% of the sample, thus exceeding interpretability criteria. Regarding model comparison tests, the LMR–LRT was significant when comparing the 2-class and 1-class models (*p* < 0.001), supporting the inclusion of a second latent class. Although the LMR–LRT remained statistically significant up to the 5-class model, the incremental improvement in fit beyond the 2-class model appeared minimal. The BLR was consistently significant (*p* < 0.001) across all models, as is commonly observed in mixture modeling. Taken together, the 2-class solution was identified as the optimal model based on four considerations: (a) substantial reductions in AIC and aBIC relative to the 1-class model, (b) high entropy indicating clear class separation, (c) sufficient class size for interpretation, and (d) greater parsimony relative to more complex alternatives.

**Figure 1:**
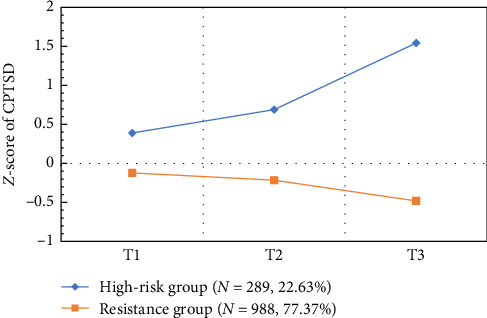
The two trajectories of CPTSD symptoms at three time points among young adults with ACEs.

Figure 1 illustrates the developmental trajectories of two subgroups of CPTSD symptoms among college students who have experienced childhood adversities.  Table 4 presents the estimated intercepts and slopes for the two identified trajectory groups. The high-risk group had a significantly higher intercept (0.45, SE = 0.09, *p* < 0.001), indicating a higher initial level of CPTSD symptoms. In contrast, the resistance group had a significantly lower intercept (−0.14, SE = 0.04, *p* < 0.001), suggesting lower baseline symptom severity. Regarding symptom change over time, the high-risk group showed a significantly positive slope (1.90, SE = 0.25, *p* < 0.001), indicating a sharp increase in CPTSD symptoms across measurement points. Conversely, the resistance group exhibited a significantly negative slope (−0.59, SE = 0.08, *p* < 0.001), reflecting a steady decline in symptom severity. These findings suggest divergent symptom trajectories between groups, with worsening in the high-risk group and recovery in the resistance group.

### 3.3. CLPNs Across Distinct CPTSD Symptom Trajectories

**Figure 2:**
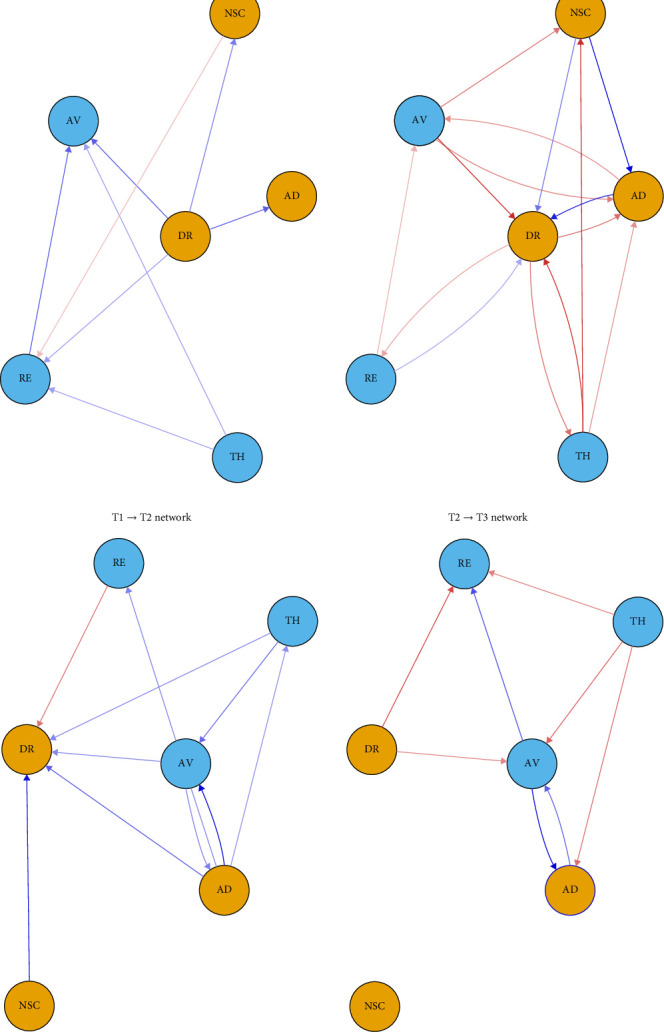
The cross-lagged panel networks for T1→T2 and T2→T3. For visualization, a beta threshold of 0.05 for the regression weights was chosen. (a) High-risk group. (b) Resistance group. RE, re-experiencing in the present; TH, sense of current threat. AD, affective dysregulation; AV, avoidance; DR, disturbed relationships; NSC, negative self-concept. The red arrows represent negative relationships, and the blue arrows indicate positive relationships.

Figure 2 shows the CLPNs from T1 to T3 in the two trajectories of CPTSD symptoms after controlling for gender, age, and lifetime traumatic exposure. From T1 to T2, the CLPNs of CPTSD symptoms revealed 12 nonzero cross-lagged edges in the high-risk group and 20 nonzero cross-lagged edges in the resistance group. From T2 to T3, there were 21 nonzero cross-lagged edges in the high-risk group and 23 nonzero cross-lagged edges in the resistance group. In the high-risk group, the relatively stronger cross-lagged edges from T1 to T2 were “disturbed relationships (DRs)→AD, AV, and NSC” and “RE in the present→AV.” The stronger cross-lagged edges from T2 to T3 were “NSC→AD,” “AD→DR,” and “AV→DR.” In the resistance group, the strongest cross-lagged edges from T1 to T2 were “NSC→DR,” “AD→AV,” and “AD→DR.” The stronger cross-lagged edges from T2 to T3 were “AV→AD,” “AV→DR,” and “sense of current TH→AV.”

**Figure 3:**
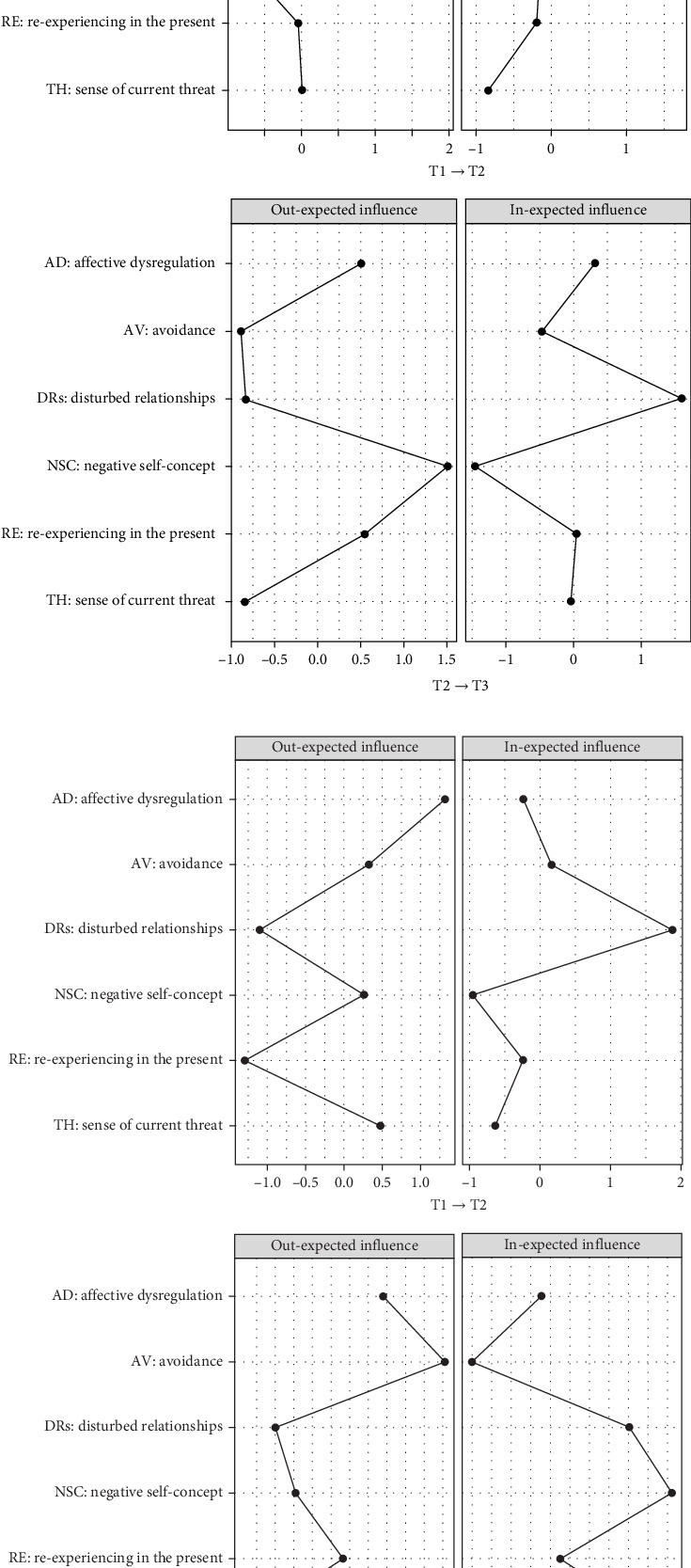
Centrality estimates in the T1→T2 and T2→T3 networks. (a) High-risk group. (b) Resistance group.

Figure 3 illustrates that, from T1 to T2, the centrality estimates revealed DR as the symptom with the highest out-EI in the high-risk group, while AD had the highest out-EI in the resistance group. For in-EI, AV ranked highest in the high-risk group, while DR had the highest out-EI in the resistance group. In the interval from T2 to T3, the centrality estimates showed that NSC had the highest out-EI in the high-risk group, whereas AV topped the out-EI in the resistance group. Similarly, DR had the highest in-EI in the high-risk group, while NSC had the highest in-EI in the resistance group.

The results of the edge weight bootstrapping program (Figure S1) demonstrate that CLPNs' estimations are moderately accurate. The results of the bootstrapping program (Figure S2) indicate that the estimations of out-EI and in-EI in both networks are generally stable. In the high-risk group, the centrality stability coefficients for both in-EI and out-EI are 0.25 and 0.25 in the T1→T2 network, while in the T2→T3 network, the coefficients for in-EI and out-EI are 0.28 and 0.13. In the resistance group, the centrality stability coefficients for both in-EI and out-EI are 0.44 and 0.44 in the T1→T2 network, while in the T2→T3 network, the coefficients for in-EI and out-EI are 0.05 and 0.25.

## 4. Discussion

By employing network modeling on longitudinal data from young adults with a history of childhood adversity, this study represents the first exploration of unique longitudinal associations among symptoms across distinct CPTSD trajectories. The analysis yielded three principal findings: First, the current study identified two distinct CPTSD trajectories: a high-risk group and a resistance group. Second, the network structures exhibited significant variation across these trajectories. In the high-risk group, DRs initially exerted the most substantial predictive influence on other CPTSD symptoms, with NSC emerging as the most predictive symptom over time. In contrast, within the resistance group, the predictive impact of DRs and NSC on other symptoms remained comparatively low, with the most predictive symptom shifting from AD to AV as time advanced.

Trajectory analysis revealed that among college students with a history of childhood adversity, CPTSD symptoms followed two distinct developmental courses over time. The high-risk group (22.63%) exhibited elevated initial symptom levels that intensified across assessments. In contrast, the resistance group (77.37%) started with lower symptom levels and showed a significant decline over time. These findings are consistent with the developmental psychopathology framework [22] and the developmental theory of PTSD [27, 28], both of which emphasize that psychological symptoms unfold through complex, time-sensitive interactions between individual and environmental factors. Although participants shared similar early adverse experiences, their symptom trajectories diverged [64]. Specifically, the high-risk group may include individuals whose developmental pathways were shaped by cumulative risk exposure, impaired self-regulatory capacities, and limited access to protective resources such as emotional or social support. In contrast, the larger resistance group may reflect the broader trend observed in most individuals with childhood maltreatment histories, namely, a capacity for at least partial psychological recovery in adulthood. This pattern supports the notion that humans, like many organisms, possess an inherent physiological and psychological propensity toward adaptation and recovery [65]. Overall, these results underscore the importance of understanding CPTSD as a dynamic condition shaped by the continuous interplay between individual vulnerability and environmental support across developmental time. However, these trajectories stand in contrast to the findings of the only other study examining CPTSD trajectories [30], which identified only level-based differences across three time points within a 6-month interval, categorizing participants into low-symptoms, moderate-symptoms, and high-risk groups. This discrepancy in findings is likely attributable to differences in the duration of the follow-up period. As a typical post-traumatic psychological response in the context of repeated and long-term interpersonal traumatic events, CPTSD symptoms typically exhibit a degree of stability [66]. Short-term assessments may be insufficient to capture the full progression of CPTSD symptoms. Therefore, long-term follow-up studies are better suited to identifying the underlying developmental patterns of the disorder.

In the CLPN model for the high-risk group, analysis of the T1→T2 network revealed that DRs exhibited the highest out-EI, indicating a strong predictive effect on other symptoms in the network, particularly AD, AV, and NSC. This result provides novel insights into the dynamic structure of CPTSD symptom networks. According to the socio-interpersonal model of PTSD [67], interpersonal disconnection following trauma may significantly disrupt emotional regulation processes and foster maladaptive coping strategies, such as AV. When individuals lack a sense of relational safety, they are deprived of essential regulatory resources that support affective stability and coherent self-processing [68]. Persistent relational dysfunction may also heighten perceptions of social TH and increase hypervigilance in interpersonal contexts. This heightened sensitivity can promote AV as an immediate strategy to reduce distress, even though it often undermines long-term adjustment [69, 70]. Furthermore, interpersonal disturbances can interfere with the development of a stable and positive self-concept. When current relational failures resemble earlier traumatic experiences, individuals may internalize these patterns, leading to persistent negative beliefs about the self [71, 72], which may contribute to the maintenance of CPTSD symptoms through recurring cycles of self-blame, shame, and emotional dysregulation. In the T2→T3 network, NSC exhibited the highest out-EI. This finding is consistent with previous studies on individuals with childhood trauma [50, 73]. The increasingly central role of NSC is theoretically consistent with models of trauma-related identity disruption, which emphasize that prolonged exposure to interpersonal trauma, particularly during developmentally sensitive periods, may undermine the formation of a stable and coherent sense of self [71, 74]. Individuals with ACEs often internalize repeated experiences of rejection, invalidation, or helplessness, leading to persistent negative beliefs about the self [74]. In addition, several strong directional associations were observed. NSC positively predicted AD, while both AD and AV positively predicted DRs. This is in line with emotion dysregulation models, which posit that maladaptive self-schemas increase individuals' susceptibility to shame, self-criticism, and emotional lability in response to interpersonal or internal stressors [75]. In turn, these emotional difficulties may lead to AV. An intriguing finding is that, as symptoms progress, the three components of DSO appear to form a mutually reinforcing cycle, whereas certain PTSD symptoms exhibit a negative predictive effect on DSO symptoms. According to Swanepoel [76], CPTSD comprises two distinct developmental pathways. PTSD symptoms tend to arise through direct neurobiological mechanisms, while disturbances in DSO emerge through more indirect routes influenced by both interpersonal and intrapersonal factors. When symptoms become severe, PTSD responses may consume core psychological resources, diminishing individuals' sensitivity to processes involved in self-awareness and interpersonal functioning. Taken together, the findings from the high-risk group highlight that the mutual reinforcement among DSO symptoms plays a pivotal role in maintaining symptom progression. Moreover, for individuals with higher symptom severity, the developmental mechanisms underlying PTSD and DSO symptoms appear to diverge.

In the CLPN model for the resistance group, we aimed to identify distinct central symptoms compared to the high-risk group, with the goal of uncovering key factors that may facilitate the transition from high to low CPTSD symptomatology. Notably, both DRs and NSC exhibited low out-EI in the resistance group, in contrast to their prominent centrality in the high-risk group. This divergence supports our hypothesis that these two symptoms may serve as critical intervention targets in early adulthood, particularly for individuals with a history of childhood adversity. Importantly, AD emerged as the most influential driver symptom in the resistance group, particularly in the T1→T2 network, where it positively predicted DRs, AV, and sense of current TH. This finding aligns with prior research indicating that AD is a central functional impairment in CPTSD and can act as a precursor to broader psychological dysfunction, even among individuals with relatively low symptom severity [77]. From the perspective of attachment theory, childhood adversity is often associated with the development of insecure or disorganized attachment patterns, which can undermine the ability to regulate emotions effectively in the face of relational or internal stressors [78]. Early disruptions in caregiving relationships may impair the development of internal working models that support emotional security and coregulation, thereby sensitizing individuals to later affective instability [79]. This is particularly relevant in early adulthood, a developmental period when autonomy and intimacy demands increase. Within this context, AD may reflect a developmental scar left by early attachment trauma and function as a central organizing symptom that shapes subsequent CPTSD trajectories. As overall symptom severity declined over time, the influence of AD on other symptoms weakened, while AV became more central in the symptom network. This shift may reflect a tendency for individuals to adopt AV as a residual coping strategy when emotional distress becomes more manageable.

It is essential to recognize the limitations of this study. First, the sample consisted exclusively of Chinese university students, which may limit the generalizability of the findings to other cultural or demographic groups. The influence of sociocultural factors on the expression, perception, and regulation of CPTSD symptoms should not be overlooked, as previous research suggests that cultural norms shape trauma responses and mental health disclosure. Therefore, future studies should aim to replicate and extend these findings in more culturally and demographically diverse populations to assess the cross-cultural validity and applicability of the symptom networks and trajectory patterns identified in this study. Second, the specific type of trauma examined may restrict the generalizability of our findings to other populations. Future studies should replicate this research using samples with diverse traumatic experiences across different cultures. Third, the centrality indices for some groups should be interpreted cautiously, as several were below 0.25 [63]. According to network analysis methodology, smaller subgroup sample sizes might contribute to lower stability of centrality indices. However, recent research suggests that sample size may not be the most critical factor influencing CS-coefficients [48]. Future research should explore the key determinants of CS-coefficient stability. Fourth, the restricted availability of the potential causal interactions between the symptoms in CLPN models. While CLPN methods are capable of identifying the directional relationships between symptoms using longitudinal data, the limitations posed by the lag time windows and the focus on a between-person level restrict the current analysis from definitively establishing “causality.” Fifth, this study only assessed common past negative life events, beyond childhood adversity, during the initial survey and did not examine recent negative life events in subsequent follow-ups. Given the heightened sensitivity to life stress among individuals with a history of childhood adversity, future research should incorporate validated measures to systematically screen for recent negative life events at each follow-up. Incorporating these events as control variables will enhance the precision and validity of the study's findings.

Despite certain limitations, these findings offer valuable clinical insights for identifying at-risk groups and understanding the dynamic relationships within different symptom networks. First, they underscore the importance of monitoring symptom evolution over time, encouraging clinicians to regularly reassess the central symptoms within each patient's CPTSD profile to refine therapeutic focus accordingly. Notably, our longitudinal network results revealed that DSO symptoms, particularly NSC and DRs, consistently predicted other symptoms across multiple time points, highlighting their central and persistent role in the maintenance of CPTSD. This suggests that, compared to PTSD alone, CPTSD is characterized by more entrenched disruptions in self-identity and interpersonal functioning, which may necessitate longer-term and more targeted therapeutic approaches. Second, given the central role of DRs in predicting other symptoms within the high-risk group, therapies such as interpersonal therapy [80] could be considered as potential interventions for individuals with high-risk CPTSD. These therapies aim to repair interpersonal relationships, address trust issues and social isolation, and ultimately enhance individuals' capacity to tolerate the distress associated with subsequent trauma-focused treatments [81]. Moreover, a NSC is also a key driving symptom among individuals at high risk for CPTSD, underscoring the importance of cognitive restructuring to challenge and modify maladaptive self-beliefs. Previous clinical evidence suggests that self-compassion training may be effective in addressing deep-seated feelings of worthlessness and failure [18]. Future research should further explore the efficacy of self-compassion-based interventions in the treatment of CPTSD.

## Figures and Tables

**Table 1:** Prevalence of ACEs and negative life events among college students with a history of childhood trauma at baseline.

Items	*N*	%
ACEs		
Physical abuse	295	23.10
Emotional abuse	150	11.75
Sexual abuse	164	12.84
Emotional neglect	221	17.31
Physical neglect	395	30.93
Parental divorce/separation	478	37.43
Mother treated violently	515	40.33
Family drug/alcohol problem	132	10.34
Family mental illness	262	20.52
Parent ever gone to prison	87	6.81
Lifetime traumatic exposure		
Natural disaster	387	30.31
Fire/explosion	193	15.11
Transportation accident	286	22.40
Serious accident at work/home/during recreational activity	275	21.53
Exposure to a toxic substance	136	10.65
Physical assault	316	24.75
Sexual assault	138	10.81
Forced captivity	114	8.93
Life-threatening illness or injury	186	14.57
Sudden, accidental death	245	19.19
Serious injury/harm/death you caused to someone else	121	9.48
Any other stressful event or experience	571	44.71

**Table 2:** Descriptive statistics and correlations among main variables.

Variables	*M* (SD)	1	2	3	4	5	6
1 Gender	—	1	—	—	—	—	—
2 Age	20.34 ± 2.80	0.09	1	—	—	—	—
3 Lifetime traumatic exposure	2.32 ± 3.34	−0.08*⁣*^*∗∗*^	−0.01	1	—	—	—
4 CPTSDT1	0.98 ± 0.84	0.06	−0.09*⁣*^*∗∗*^	0.19*⁣*^*∗∗∗*^	1	—	—
5 CPTSDT2	0.72 ± 0.79	−0.04	−0.06*⁣*^*∗*^	0.16*⁣*^*∗∗∗*^	0.39*⁣*^*∗∗∗*^	1	—
6 CPTSDT3	0.64 ± 0.80	−0.09*⁣*^*∗∗*^	−0.07*⁣*^*∗∗*^	0.05	0.27*⁣*^*∗∗∗*^	0.41*⁣*^*∗∗∗*^	1

*⁣* ^*∗*^ *p* < 0.05.

*⁣* ^*∗∗*^ *p* < 0.01.

*⁣* ^*∗∗∗*^ *p* < 0.001.

**Table 3:** Model fit for the different growth mixture models.

Model	AIC	aBIC	Entropy	LMR–LRT (*p*)	BLRT (*p*)	Smallest group
1C	10424.248	10440.054	—	—	—	—
**2C**	**9941.548**	**9963.281**	**0.897**	**0.000**	**0.000**	**303 (22.63%)**
3C	9915.430	9943.091	0.831	0.023	0.000	63 (4.93%)
4C	9761.000	9794.588	0.846	0.052	0.000	141 (11.04%)
5C	9376.722	9416.237	0.955	0.000	0.000	3 (0.23%)
6C	9382.722	9428.165	0.960	0.003	0.000	0 (0%)

*Note*. Bold type indicates the final cluster solution.

Abbreviations: aBIC, adjusted Bayesian Information Criterion; AIC, Akaike Information Criterion; BIC, Bayesian Information Criterion; BLRT, bootstrapped likelihood ratio test; LMR–LRT, Lo–Mendell–Rubin likelihood ratio test.

**Table 4:** Statistics for the selected two-group model.

Group	Parameter estimate (standard error)	
	Intercept	Slope
High-risk group	0.45 (0.09)*⁣*^*∗∗∗*^	1.90 (0.25)*⁣*^*∗∗∗*^
Resistance group	−0.14 (0.04)*⁣*^*∗∗∗*^	−0.59 (0.08)*⁣*^*∗∗∗*^

*⁣* ^*∗∗∗*^ *p* < 0.001.

## Data Availability

The data supporting this study's findings are available from the corresponding author upon reasonable request.

## Supporting Information

Supporting Information Descriptive statistics of each symptom at three time points in a two-group model are shown in Supporting Information Table S1. Supporting Information Figure S1 provides the results for the accuracy of the networks. Supporting Information Figure S2 provides the results for the stability of centrality measures in networks.
