# Supplementary material for: Cross-Lagged Panel Networks of Distinct Complex Post-Traumatic Stress Disorder Symptom Trajectories Among Young Adults With Adverse Childhood Experiences
Source: Depress Anxiety. 2025 Sep 16;2025:8823021. doi: 10.1155/da/8823021 (PMC12457065; doi:10.1155/da/8823021)
Supplement: Supporting Information — Descriptive statistics of each symptom at three time points in a two-group model are shown in Supporting Information Table S1. Supporting Information Figure S1 provides the results for the accuracy of the networks. Supporting Information Figure S2 provides the results for the stability of centrality measures in networks. [file 8823021.f1.docx]

Table S1 Descriptive statistics of each symptom at three time points in a two-group model

|  | High-risk group | | | Resistance group | | |
| --- | --- | --- | --- | --- | --- | --- |
| Nodes | T1 | T2 | T3 | T1 | T2 | T3 |
| Re-experiencing in the present | 1.11 (1.06) | 1.13 (1.10) | 1.81 (0.93) | 0.75 (0.94) | 0.58 (0.87) | 0.28 (0.57) |
| Avoidance | 1.39 (1.33) | 1.35 (1.25) | 2.06 (0.94) | 0.86 (1.09) | 0.60 (0.94) | 0.26 (0.55) |
| Sense of current threat | 1.16 (1.16) | 1.08 (1.10) | 1.76 (0.92) | 0.67 (0.67) | 0.40 (0.72) | 0.18 (0.44) |
| Affective dysregulation | 1.38 (1.24) | 1.35 (1.19) | 1.87 (0.96) | 0.86 (0.94) | 0.54 (0.87) | 0.27 (0.66) |
| Negative self-concept | 1.32 (1.28) | 1.25 (1.13) | 1.96 (0.94) | 0.75 (0.85) | 0.46 (0.79) | 0.21 (0.58) |
| Disturbed relationships | 1.59 (1.18) | 1.43 (1.08) | 2.09 (0.79) | 1.20 (1.20) | 0.76 (0.90) | 0.42 (0.45) |

*Note:* The values in parentheses represent the standard deviation, while the values to the left of the parentheses represent the mean.

1. High-risk group


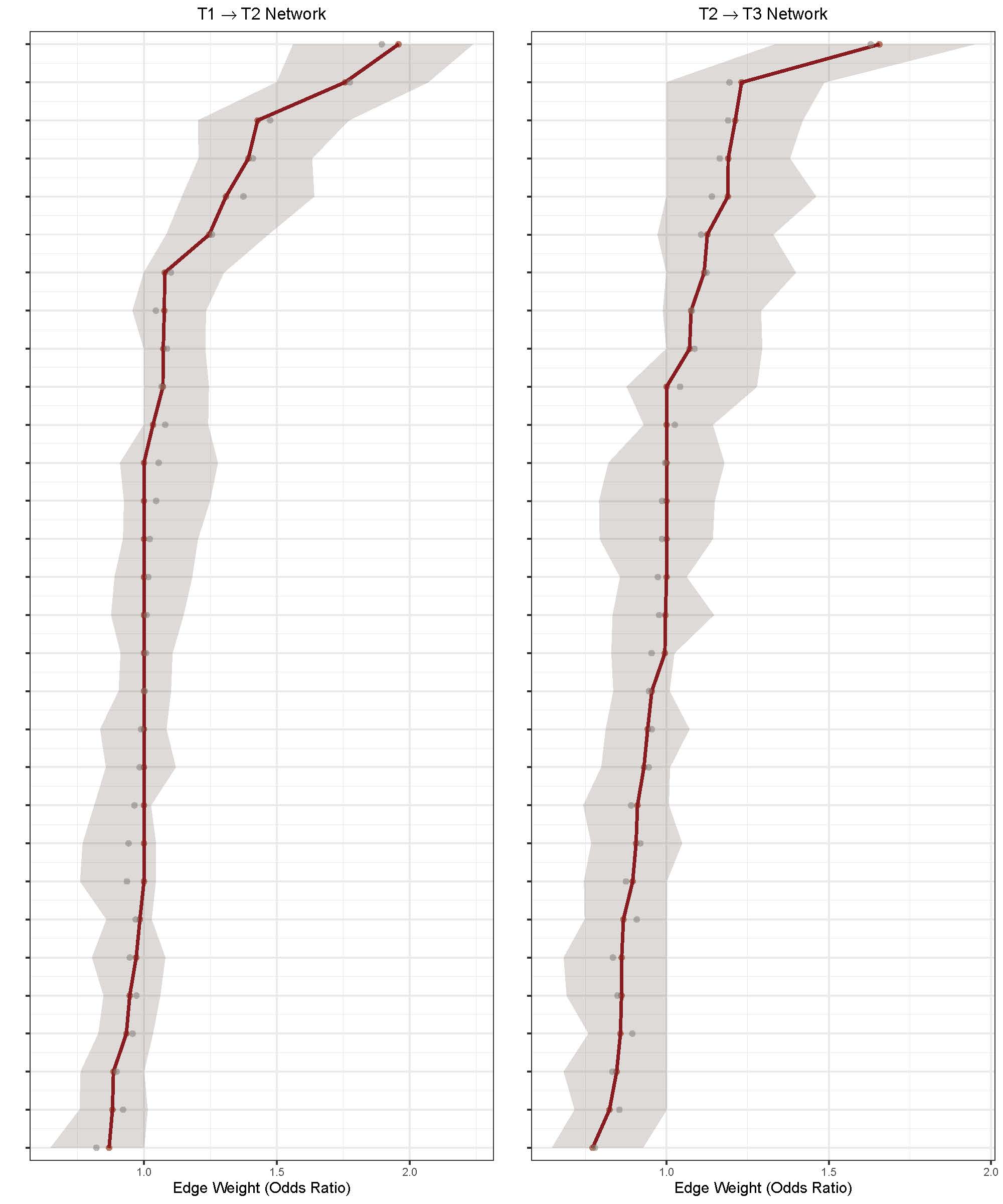


1. Resistance group


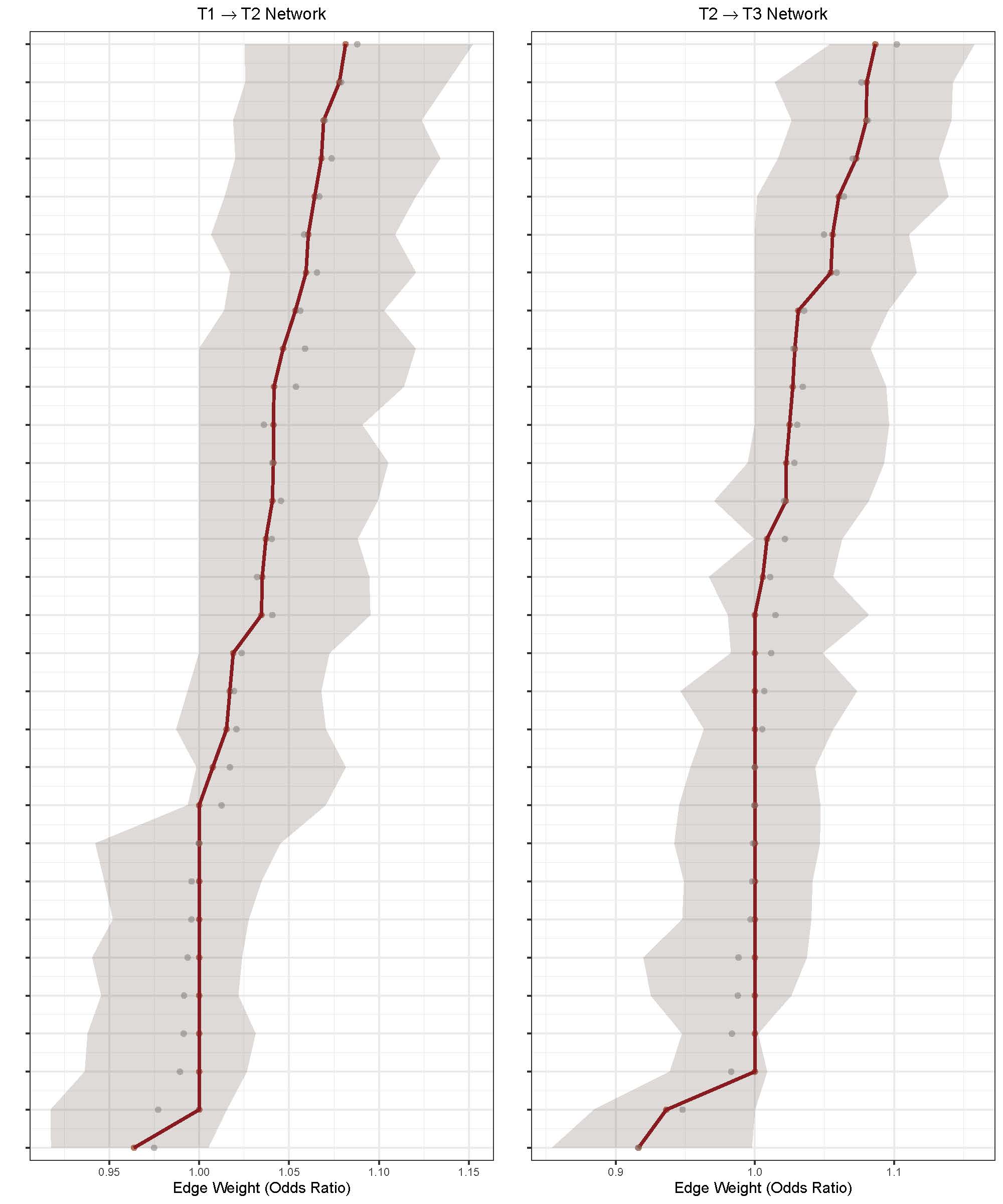


**Figure S1** Edge-weight accuracy for networks in high-risk and resistance group. Bootstrapped confidence intervals of estimated edge-weights for the estimated network. The red line indicates the sample values and the gray area indicates the bootstrapped confidence intervals. Each horizontal line represents one edge of the network, ordered from the edge with the highest edge-weight to the edge with the lowest edge-weight.

1. High-risk group


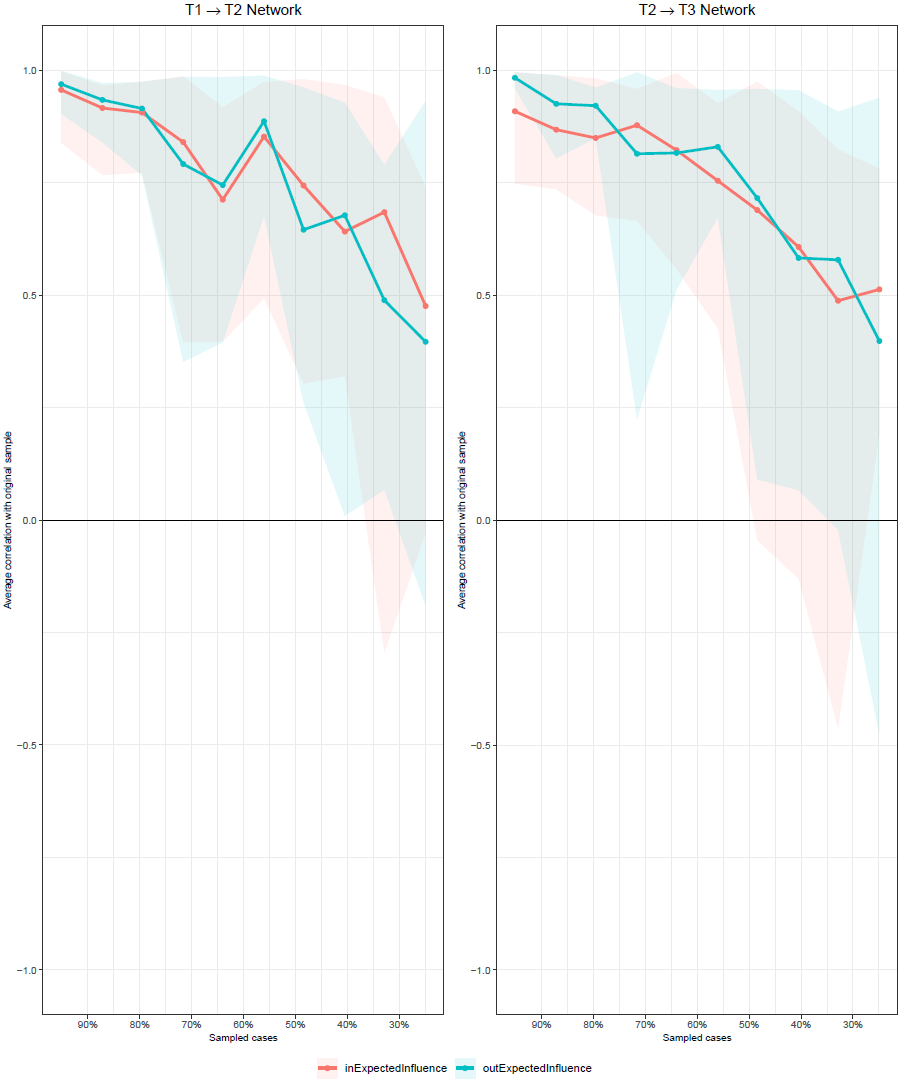


(b) Resistance group


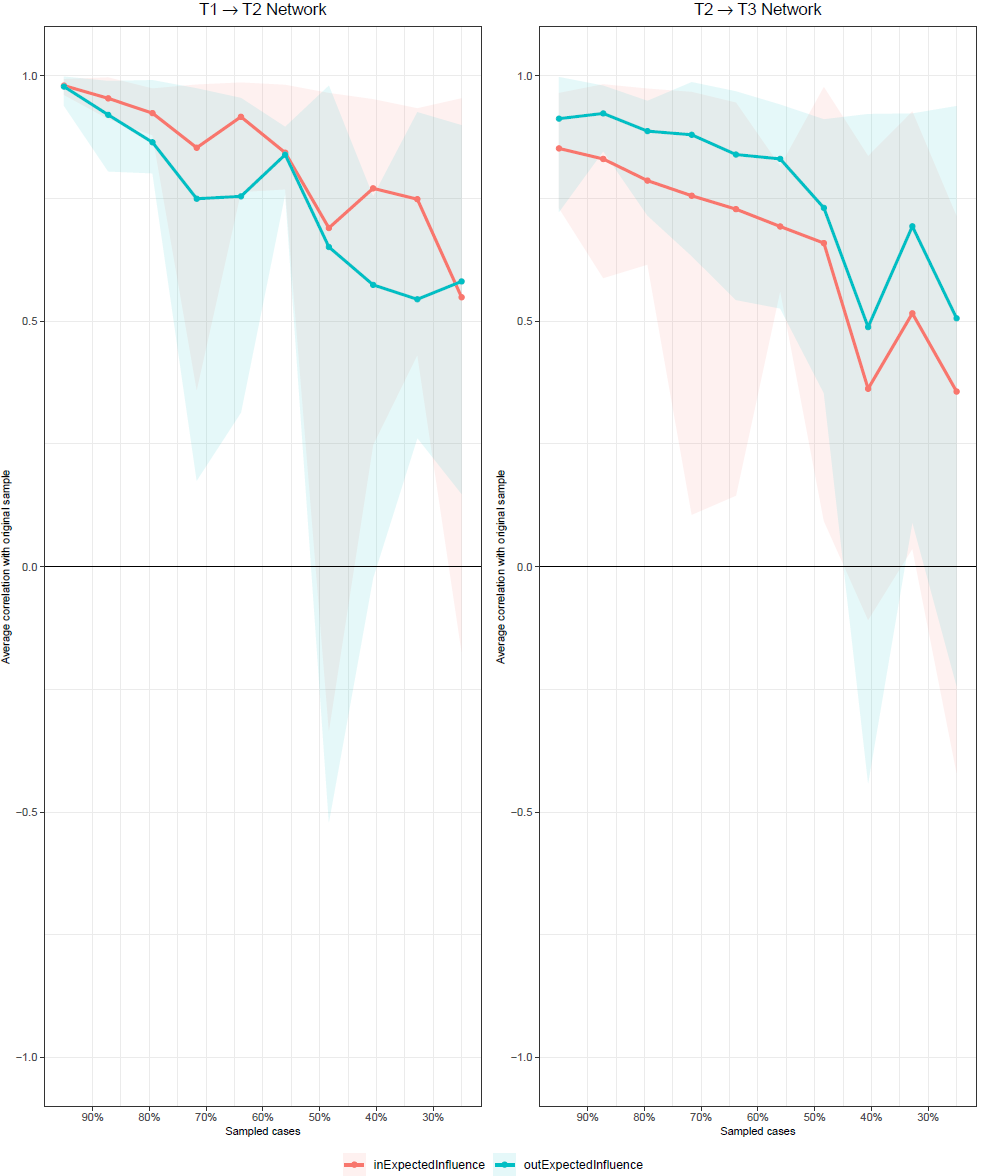


**Figure S2.** Stability of centrality measures in CLPN.
